# The bicipital groove as a landmark for reconstruction of complex proximal humeral fractures with hybrid double plate osteosynthesis

**DOI:** 10.1186/s12893-016-0125-6

**Published:** 2016-03-12

**Authors:** Jan Theopold, Bastian Marquaß, Johannes Fakler, Hanno Steinke, Christoph Josten, Pierre Hepp

**Affiliations:** Department of Orthopedics, Trauma and Plastic Surgery, University of Leipzig, Liebigstrasse 20, 04103 Leipzig, Germany; Institute of Anatomy, University of Leipzig, Liebigstrasse 13, 04103 Leipzig, Germany

**Keywords:** Proximal humerus fracture, Metaphyseal comminution, Double plate, Locked plate

## Abstract

**Background:**

Complex proximal humerus fractures with metaphyseal comminution remain challenging regarding reduction and stability. In most fracture patterns the hard bone of the bicipital groove remains intact. In this case series, we describe a novel technique of hybrid double plate osteosynthesis of complex proximal humerus fractures with metaphyseal comminution.

**Methods:**

In randomly chosen shoulder specimens and synthetic bones, pilot studies for evaluation of the feasibility of the technique were performed. Between 4/2010 and 1/2012 10 patients underwent hybrid double plate osteosynthesis. Seven patients (4 male, 3 female, mean age was 50 years (range 27–73)) were available for retrospective analysis. Based on plain radiographs (anterior-posterior and axial view), the fractures were classified according to the Orthopaedic Trauma Association classification (OTA) and by descriptive means (head-split variant (HS), diaphyseal extension or comminution (DE)).

**Results:**

Follow-up radiographs demonstrated complete fracture healing in six patients and one incomplete avascular necrosis. None of the patients sustained loss of reduction. Three patients where reoperated. The medium, not adapted, Constant score was 80 Points (58–94). Patients subjective satisfaction was graded mean 3 (range: 0–6) in the visual analog scoring system (VAS).

**Conclusion:**

The technique of hybrid double plate osteosynthesis using the bicipital groove as anatomic landmark may re-establish shoulder function after complex proximal humerus fractures in two dimensions. Firstly the anatomy is restored due to a proper reduction based on intraoperative landmarks. Secondly additional support by the second plate may provide a higher stability in complex fractures with metaphyseal comminution.

## Background

Complex proximal humerus fractures with metaphyseal extension or comminution are challenging regarding reduction and stability of the osteosynthesis [1, 2]. The metaphysis may be broken into several fragments and anatomical landmarks for reduction may be absent. In addition there will not be a sufficient metaphyseal substance to provide support for fixation. The missing restoration of the medial cortical support has been identified being responsible for an increased failure rate [1, 2] and nonunions [3] of the proximal Humerus. The initial calcar comminution is an independent prognostic factor for bad clinical outcome [1, 2]. This may lead to secondary varus dislocation with biomechanical alteration due to decreased supraspinatus efficiency [4]. Whereas locking plates with placement of calcar screws [2, 5] and locking plates in combination with fibular bone grafts [6, 7] have been described to increase the medial support, hybrid double-plating has not been considered for improving stability, yet.

In this report, we describe a technique of double plate osteosynthesis of complex proximal humerus fractures with metaphyseal comminution. In the first step the bicipital groove is used as an anatomical landmark for reduction of the anterior column and a one-third tubular plate is placed into the groove in an inverted fashion. Secondly the reduction is completed and the fracture is fixed by a laterally placed locking plate.

## Methods

**Fig. 1:**
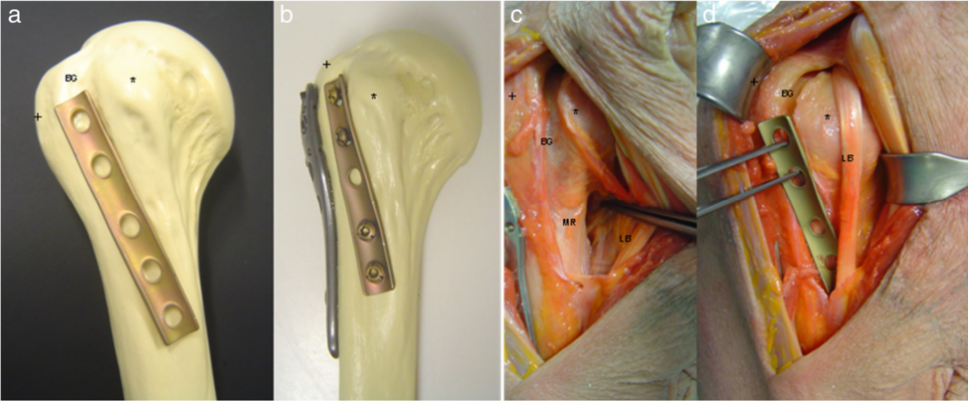
Basic principles of hybrid double plating. **a** the tubular plate fits into the bicipital groove in inverted fashion. **b** the locking plate (here: WinstaPH, Axomed, Freibung, Germany) is fixed laterally, **c** anatomy in a cadaver specimen showing exposure if the metaphyseal region, **d** the tubular plate is adjusted into the groove (+ greater tuberosity, * lesser tuberosity, BG bicipital groove, LB tendon of the long head of the biceps, MR metaphyseal region)

In randomly chosen shoulder specimens (donors gave directed consent that their cadavers were to be used in teaching or for research projects at the Institute of anatomy) and synthetic bones, pilot studies for evaluation of the feasibility of the technique were performed (Fig. 1) The double plate osteosynthesis involves a one third tubular plate placed into the bicipital groove in an inverted fashion. The locking plate is fixed laterally in classical fashion.

**Fig. 2:**
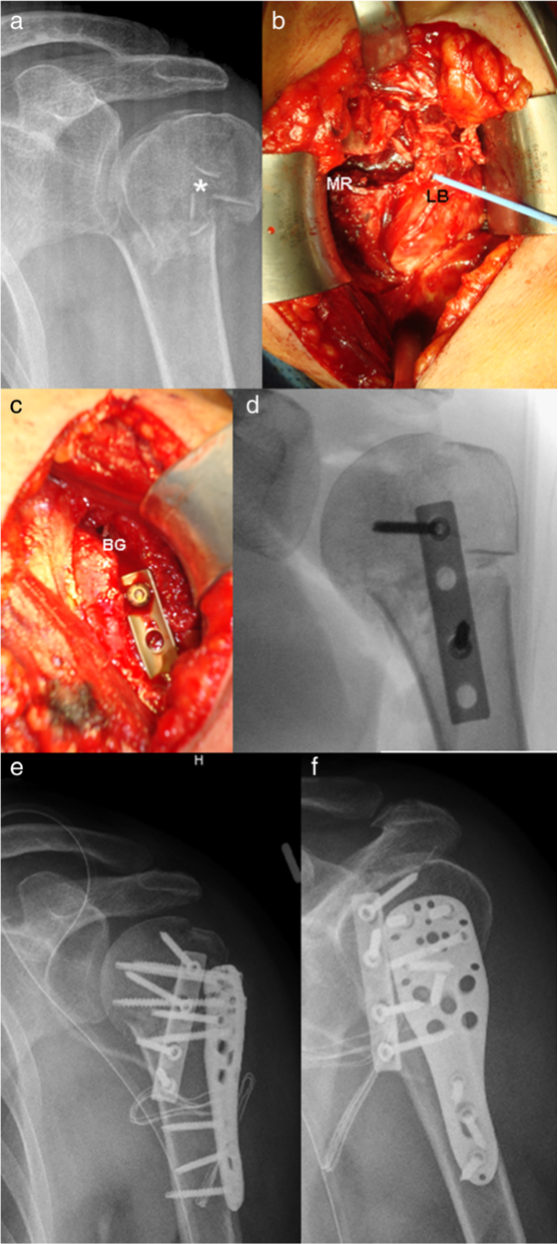
**a** a.p. view of a proximal humerus fracture with metaphyseal comminution (*) **b** intraoperative view showing the comminuted zone; long biceps tendon with loop (LB), metaphyseal region (MR) **c**, **d** the reduction is accomplished with the inverted tubular plate within the sulcus of the biceps tendon; bicipital groove (BG) **e**, **f** postoperative a.p. and y- radiograph with double plate configuration

The patient is placed in beachchair position and positioned such that anteroposterior and axillary views of the proximal humerus can be easily obtained using C-arm fluoroscopy. The fracture is exposed through a deltopectoral approach. The long biceps tendon is identified and followed proximally. The rotator interval is partially opened and a biceps tendon tenotomy is performed. Now the anterior aspect of the fracture including parts of the medial metaphysical region can be visualised and analysed (Fig. 2b). Fracture reduction is performed by standard methods, including Kirschner wire and elevators to control the humeral head fragment. A one third tubular plate is placed into the groove in an inverted fashion and fixed to the proximal part with 2 screws. Further reduction is achieved by fixing the plate distally (Fig. 2c, d). The pectoralis major tendon may be partially released from the humeral shaft to achieve sufficient overview. Further reduction of the fracture is accomplished (e.g. greater tuberosity) and the locking plate is applied laterally (Fig. 2e, f). Special attention should be paid to the position of the implant. Once the fracture has been provisionally reduced and the implant temporarily fixed with Kirschner wires, the reduction and implant placement should be verified on C-arm fluoroscopy. Depending on the patient’s age a soft tissue tenodesis of the biceps tendon is performed beside the tubular plate.

After surgery patients had individual patient-related postoperative management. For better comfort, the patient’s arm was placed in a sling for a maximum of 2 weeks. Passive and active range of motion exercises were started after surgery, depending on pain and activity level.

### Patients

**Fig. 3:**
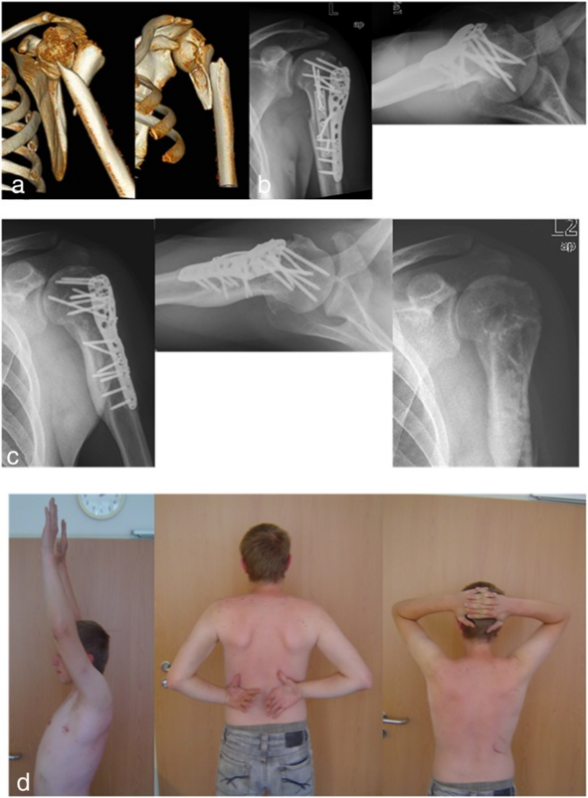
**a** 24 year old male with dislocated proximal fracture with metaphyseal comminution and intermuscular fragment. **b** postoperative x-ray showing anatomical reduction after double plate osteosynthesis. **c** X-ray after one year showing complete healing of the fracture, x-ray after material removal. **d** functional result after 2 years, Constant 90 points

Between 4/2010 and 1/2012 10 patients underwent hybrid double plate osteosynthesis. All patients were treated by one surgeon (P.H.). In the present case series, the indication for double-plating was individual and depending on intraoperative reduction. Retrospective review of patient records and radiographs was approved by the institutional review board of the medical faculty of the University of Leipzig, Germany (no. 062-14-10032014). All included patients gave their informed consent. Consent to publish Fig. 3 and the patient information contained in Table1was also obtained. Three patients were excluded (one died, one was lost to follow-up and one patient sustained a pathological fracture due to a mamma cancer). Thus, seven patients (4 male, 3 female, mean age was 50 years (range 27–73)) were available for retrospective analysis. Based on plain radiographs (anterior-posterior and axial view), the fractures were classified according to the Orthopaedic Trauma Association classification (OTA) and by descriptive means (head-split variant (HS), diaphyseal extension or comminution (DE)).

**Table 1:** Overall characteristics and results of the seven patients with minimum 12 months follow-up

Case no.	Age	Sex	OTA proximal segment classification	Fracture morphology	Follow-up (months)	Constant score (affected/unaffected side)	Shoulder range of motion				Cause of injury	Complications/Reoperation
							Flexion	Abduction	Internal rotation	External rotation		
1	24	M	11-B2.3	DE	13	94/96	170	180	90	90	MVA	Material removal after 12 months
2	65	F	11-C2.2	DE, HS	35	72/93	160	90	90	50	F	
3	73	M	11-C2.1	DE, HS	34	79/85	160	150	90	50	F	
4	39	F	11-C2.1	DE, HS	28	85/93	170	160	90	50	BA	AVN, Screw perforation, frozen shoulder, 2 reoperations
5	36	M	11-B2.3	DE	18	84/93	150	150	40	60	FFH	
6	63	M	11-C2.1	DE, HS	26	91/94	160	130	90	60	BA	
7	51	F	11-B2.3	DE	24	58/84	110	100	90	50	F	Material removal after 13 months

*F* female, *M* male, *MVA* motor vehicle accident, *BA* bicycle accident, *FFH* fall from high, *F* fall, *DE* proximal humerus fracture with diaphyseal extension or diaphyseal comminution, *HS* head-split variant, *AVN* avascular head necrosis

## Results

Table 1 summarizes the demographic, injury and follow up data for each patient in our series. Follow-up radiographs demonstrated complete fracture healing in six patients and one incomplete avascular necrosis. None of the patients sustained loss of reduction. Three patients where reoperated: Two elective material removal after one year (Fig. 3) and one removal of perforating screws after 9 months and complete material removal with arthrolysis of the shoulder joint after 21 months. The medium, not adapted, Constant score is 80 Points (58–94). The medium Constant score of the non-affected side was 91 Points (84–96). Functional results revealed a mean range of motion (flexion/extension/adduction/abduction/internal rotation, external rotation) of 150 (110–170)/40 (30–50)/40 (30–50)/140 (90–180)/60 (50–90)/and 80 (40–90)°. Patients subjective satisfaction was graded mean 3 (range: 0–6) in the visual analog scoring system (VAS).

## Discussion

Taking into account that adequate reduction and stable conditions in osteosynthesis of proximal humerus fractures may be crucial for preservation and revascularization of the humeral head [8], the double plate osteosynthesis with consideration of the bicipital groove as anatomical landmark has been developed to restore the anatomy and optimize the stability after complex proximal humerus fractures.

Double plating with two one-third tubular plates in 90° configuration has previously been described in clinical studies providing high stability and allowing early mobilization [9]. On the other hand biomechanical studies pointed out superior biomechanical properties of locking plates when compared to double plate with one-third tubular plates [10]. Whereas hybrid plating – the combination of locking and nonlocking screws in the same plate – has been shown to provide an attractive alternative to an all-locked construct [11], to our knowledge the combination of a locking plate with nonlocking tubular plate has not been described, yet. This hybrid configuration of the one-third tubular plate anatomically fitting in the bicipital groove and the advantages of the locking plate in osteoporotic bone may lead to higher strength of the construct.

Whereas the bicipital groove is used as an anatomical landmark to restore humeral head retroversion when treating complex proximal humeral fractures with arthroplasty [12, 13], it has not been taken into account as landmark for the reconstruction with plate osteosynthesis. In most fracture patterns the hard bone of the bicipital groove remains intact [14]. The inverted one-third tubular plate fits into this sulcus. Nevertheless anatomical variations have to be taken into account [15] and the tubular plate may not always optimally fit. One may criticize the tenotomy of the biceps tendon for exposure of the sulcus. On the other hand the tendon has recently been identified as potential source for pain in complex proximal humerus fractures [16] and is routinely performed in hemiarthroplasty. In a series of arthroscopic material removal 30 % tenotomies were performed due to biceps tendon pathologies after proximal humerus fractures [17]. In our experience the biceps tendon tenotomy provides a greater exposure of the metaphyseal comminution zone, enables a distinct visualisation and palpation of the joint surface through the rotator interval and this may potentially reveal hidden head-split variants.

Limitations: Our study also has the same inherent weakness that is seen in many other retrospective studies. One concern may be the lack of any controls. In order to achieve a desired test power of 0.95, with an expected effect size d of 0.5 a total sample size of n = 176 (88 per group) would have been required for patients and controls.

*In our series, the patients treated with double plate technique sustained complex proximal humerus fracture. Retrospectively, 57 % (n = 4) showed a head-split variant in combination with the metaphyseal comminution. In the literature this kind of fracture are often treated with arthroplasty* [18, 19]*. Except of one patient with persisting pain and functional impairment, the overall Constant score was predominately excellent and good. The functional results were comparable with current literature* [8, 20]*.*

*One further limitation of the presented case series is the lack of a reliable algorithm for the use of double plate osteosynthesis. The decision making was individual in each case and was performed, when reduction or stability was subjectively unsatisfying.*

## Conclusion

In conclusion, the technique of hybrid double plate osteosynthesis using the bicipital groove as anatomic landmark may re-establish shoulder function after complex proximal humerus fractures in two dimensions. Firstly the anatomy is restored due to a proper reduction based on intraoperative landmarks. Secondly additional support by the second plate may provide a higher stability in complex fractures with metaphyseal comminution. Ultimately, comparative biomechanical evaluations and long term follow-up studies in vivo are required to delineate the advantages.
